# Supplementary material for: Molecular profiling of brain endothelial cell to astrocyte endfoot communication in mouse and human
Source: Nat Commun. 2025 Nov 6;16:9750. doi: 10.1038/s41467-025-65487-4 (PMC12592424; doi:10.1038/s41467-025-65487-4)
Supplement: Supplementary file 1 — Supplementary Information [file 41467_2025_65487_MOESM1_ESM.pdf]

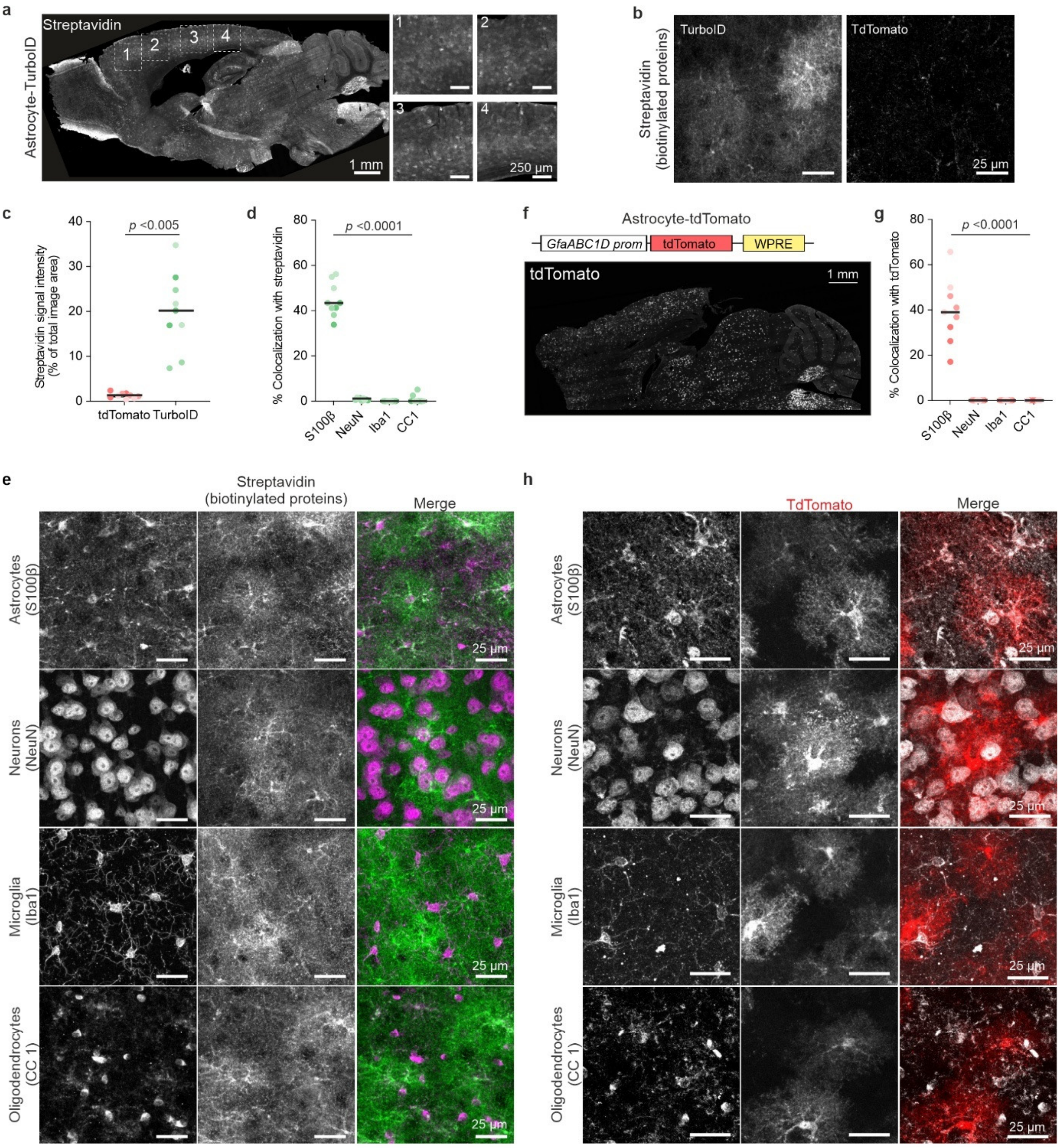

Supplementary figure 1

Supplementary fig. 1. Assessment of Astrocyte-TurboID and Astrocyte-TdTomato AAV expression. a: Image of the brain wide streptavidin signal after expression of Astrocyte-TurboID AAV and biotin injections (Fig. 1c). Insets 1-4 show expression throughout the cortex. Scale bar = 1 mm for a, 250 μm for Insets 1-4. **b:** Representative images of streptavidin signal in TurboID and tdTomato-injected brains. Scale bar = 25 μm. **c:** Streptavidin signal intensity in TurboID and tdTomato-injected brains. A linear mixed-effects model (LMM) was used to account for repeated measures from each mouse: Intensity ~ AAV + (1|Mouse), N = 3 mice per AAV, 3 images per mouse; $F(1,4) = 32.70$. In each graph, black horizontal lines represent the median, and data points from different mice are represented with different shades of red (tdTomato) and green (TurboID). **d:** Cell-specific quantification of streptavidin-positive cells. From 360 S100β cells counted, 152 were streptavidin positive. LMM was used to account for repeated measures from each mouse: Percent_Streptavidin ~ Marker + (1|Mouse), N = 3 mice per marker, 3 images per mouse; $F(3,30) = 330.9$ with Tukey post hoc tests. Black horizontal lines represent the median, and data points from different mice are represented with different shades of green. **e:** Presence of biotinylated proteins in astrocytes (S100β), neurons (NeuN), microglia (Iba1), and oligodendrocytes (CC1). Scale bars = 25 μm. **f:** Image of the brain wide RFP fluorescence signal after expression of Astrocyte-tdTomato AAV. Scale bar = 1 mm. **g:** Cell-specific quantification of tdTomato-positive cells. From 345 S100β cells counted, 134 were tdTomato positive. LMM was used to account for repeated measures from each mouse: Percent_TdTomato ~ Marker + (1|Mouse), N = 3 mice for S100β, NeuN, Iba1, N = 2 mice for CC1; 3 images per mouse; $F(3,27.66) = 69.49$ with Tukey post hoc tests. Black horizontal lines represent the median, and data points from different mice are represented with different shades of green. **h:** Presence of tdTomato expression in astrocytes (S100β), neurons (NeuN), microglia (Iba1), and oligodendrocytes (CC1). Scale bars = 25 μm. Source data are provided as a Source Data file.

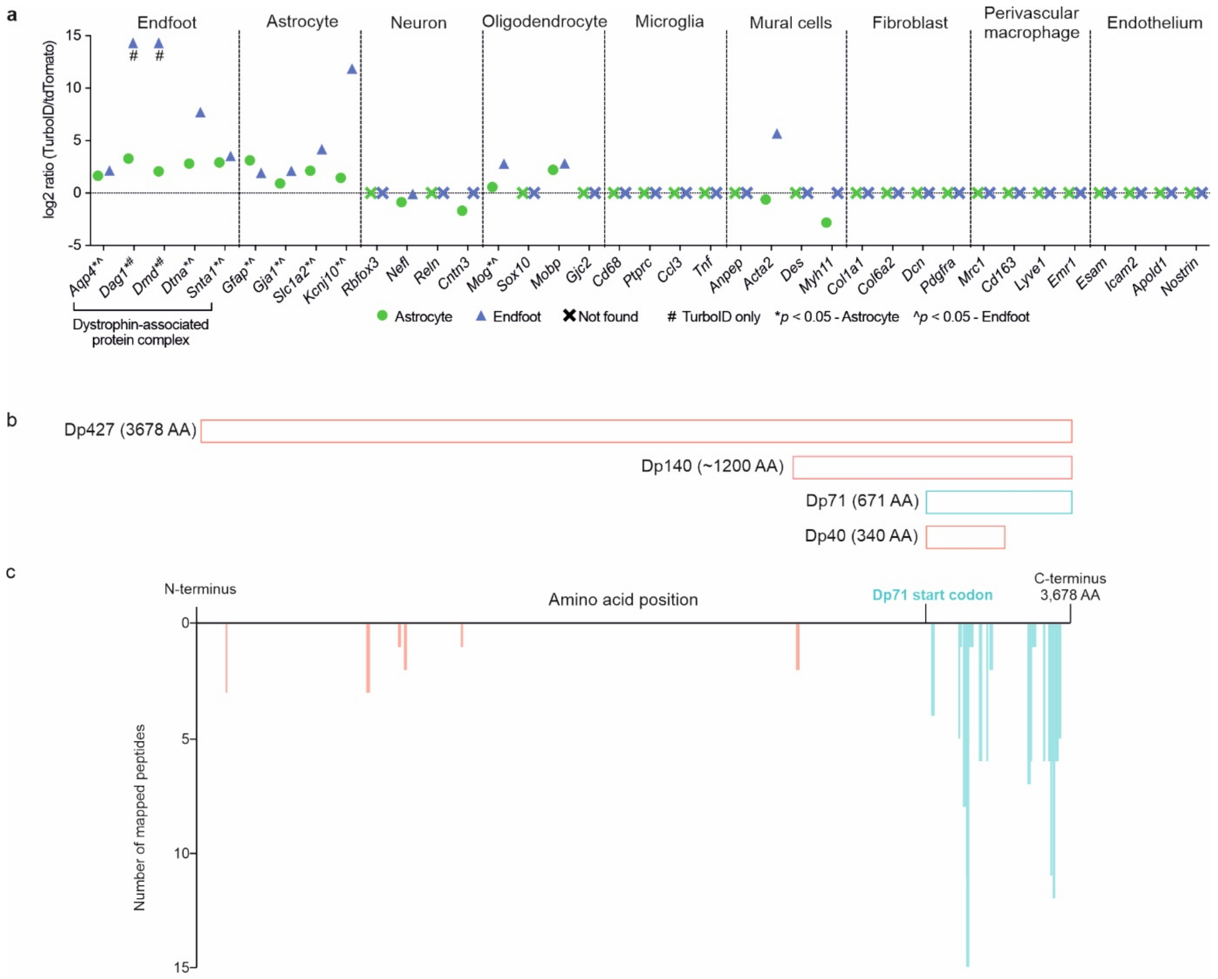

Supplementary figure 2

__Supplementary fig. 2.__ **Analysis of the endfoot proteome specificity. a:** Cell-specific protein abundance, derived from LC-MS/MS data, expressed as $\log_2$(TurboID/tdTomato) for astrocyte (green circles) and endfoot (blue triangles). Green and blue X indicate that the protein was not found in the astrocyte or endfoot datasets, respectively. * and ^ indicates proteins that are significantly enriched in Astrocyte- and Endfoot-TurboID sample types versus their tdTomato controls, respectively. # indicates proteins not found in Endfoot-tdTomato, so relative enrichment cannot be calculated. **b:** Representative diagrams of common dystrophin (DMD) isoforms expressed in brain and their amino acid (AA) lengths. Full-length DMD (Dp427) is 3,678 amino acids in mouse. Isoform Dp140 is not annotated in mouse, but is ~1,225 AA long in human, and assumed to be ~1,200 AA long in mouse (https://www.uniprot.org/uniprotkb/A0A5H1ZRQ1/entry). **c:** Graph of peptides in the endfoot proteome mapped to DMD. Each line represents a specific peptide; the width of the line represents the length of the peptide, and the length of the line represents the number of times the peptide was found. Peptides aligning with the Dp71 isoform (enriched in endfeet) are colored in teal. Source data are provided as a Source Data file.

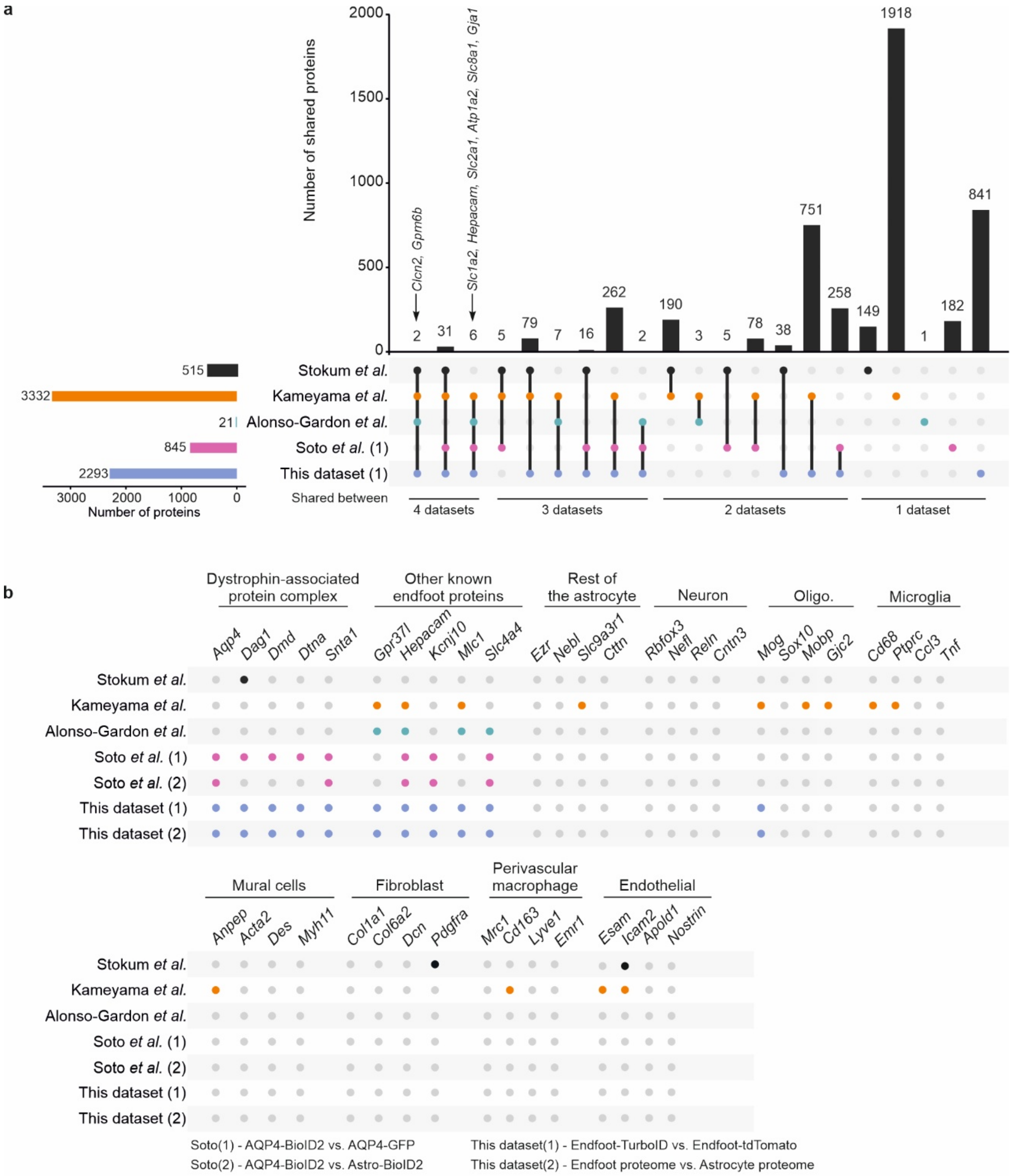

Supplementary figure 3

Supplementary fig. 3. Comparison of the endfoot proteome described in this manuscript with previously published endfoot datasets. **a**: UpSet plot comparing our endfoot proteome (This dataset, blue) with other endfoot proteome attempts[36-39] (Stokum *et al.*, whole brain, black dots; Kameyama *et al.*, whole brain, orange dots; Alonso-Gardon *et al.*, whole brain, teal dots; Soto *et al.*, striatum, pink dots). The bars indicate the number of shared proteins between the datasets indicated by the connected dots below. The total number of proteins in each dataset is plotted in horizontal bar graphs. **b**: Dot plot illustrating the presence of the endfoot-enriched dystrophin-associated protein complex, other known endfoot proteins, astrocyte enriched proteins that are depleted in endfeet, and cell-specific proteins for neurons, oligodendrocytes, microglia, mural cells, fibroblasts, perivascular macrophages and BECs. Total proteins identified for each dataset: Stokum *et al.*, 515 proteins; Kameyama *et al.*, 3332 proteins; Alonso-Gardon *et al.*, 21 proteins; Soto *et al.* (1), 845 proteins; Soto *et al.* (2), 658 proteins; This dataset (1), 2293 proteins; This dataset (2), 1867 proteins. For data from Soto *et al.*, (1) indicates AQP4-BioID2 vs. AQP4-GFP and (2) indicates AQP4-BioID2 vs. cytoplasmic Astro-BioID2. For data from This Dataset, (1) indicates Endfoot-TurboID vs. Endfoot tdTomato and (2) indicates Endfoot proteome vs. Astrocyte proteome. Source data are provided in Supplementary Data 3.

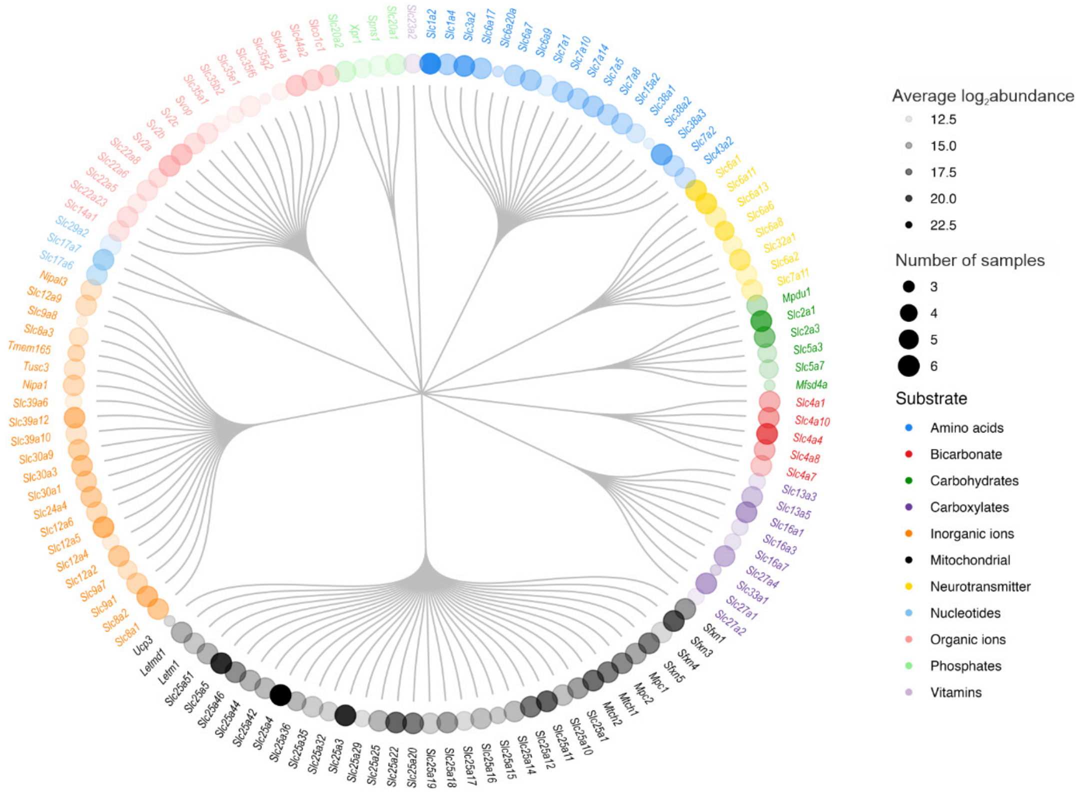

Supplementary figure 4

Supplementary fig. 4. Circular dendrogram of the solute carrier family proteins (SLCs) identified in the endfoot proteome. Branches of the tree and color separate proteins based on the type of transporter. The color intensity of each dot corresponds to protein abundance, while the size of the dot corresponds to the number of samples in which the protein was identified (total N = 6 mice). Proteins are referred to by their gene names. Source data are provided in Supplementary Data 2.

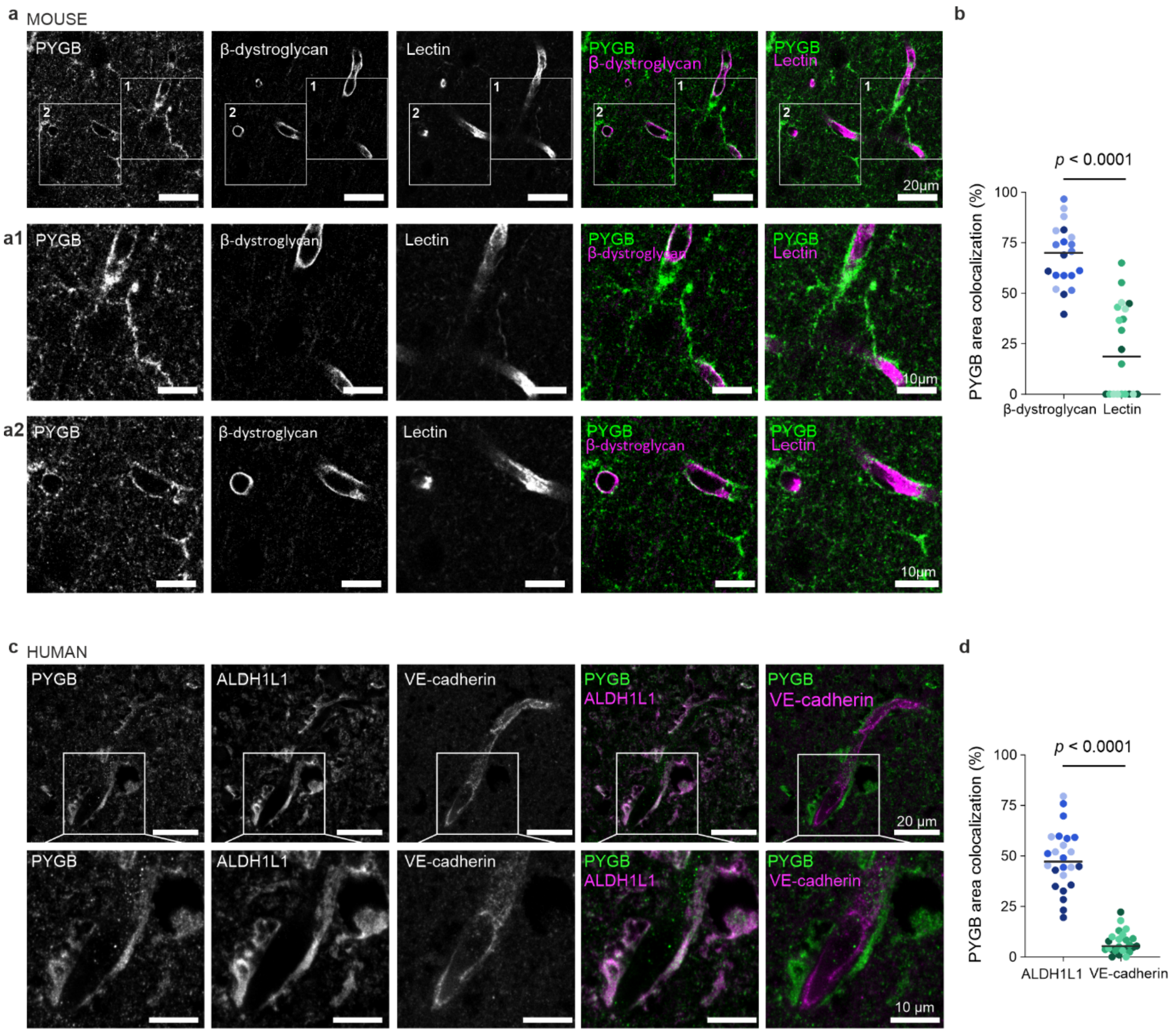

Supplementary figure 5

Supplementary fig. 5. Validation of PYGB localization in astrocyte endfeet. a: Representative immunofluorescence image of PYGB (green), endfeet labeled with β-dystroglycan (magenta), and vessels with lectin (magenta) in mouse cortex. White squares indicate regions shown in a1 and a2. For a scale bar = 20 μm, and a1-a2 scale bar = 10 μm. **b:** Quantification of PYGB colocalization with β-dystroglycan or lectin. Data are shown as individual values representing the % of total β-dystroglycan or lectin area that is colocalized with PYGB. Horizontal lines indicate the median, data points from the same mouse are represented with different color shades. LMM was used to account for repeated measures from each mouse: %Area ~ Marker + (1 | Animal), N = 4 mice, 5 vessel cross-sections per mouse; $F(1,35) = 66.76$. **c:** Representative immunofluorescence images from post-mortem human brain frontal cortex (BA46) immunolabeled with PYGB (green), ALDH1L1 (magenta) and VE-cadherin (magenta). Scale bar = 20 μm. The white squares indicate the region of each image displayed at higher magnification below. Scale bar = 10 μm. **d:** Quantification of PYGB colocalization with ALDH1L1 or VE-cadherin. Data are shown as individual values representing the % of total ALDH1L1 or VE-cadherin area that is colocalized with PYGB. LMM was used to account for repeated measures from each case: %Area ~ Marker + (1 | Case), N = 3 cases, 8 vessels per case; $F(1,44) = 185.37$. Horizontal lines indicate the median, data points from the same case are represented with different color shades. Source data are provided as a Source Data file.

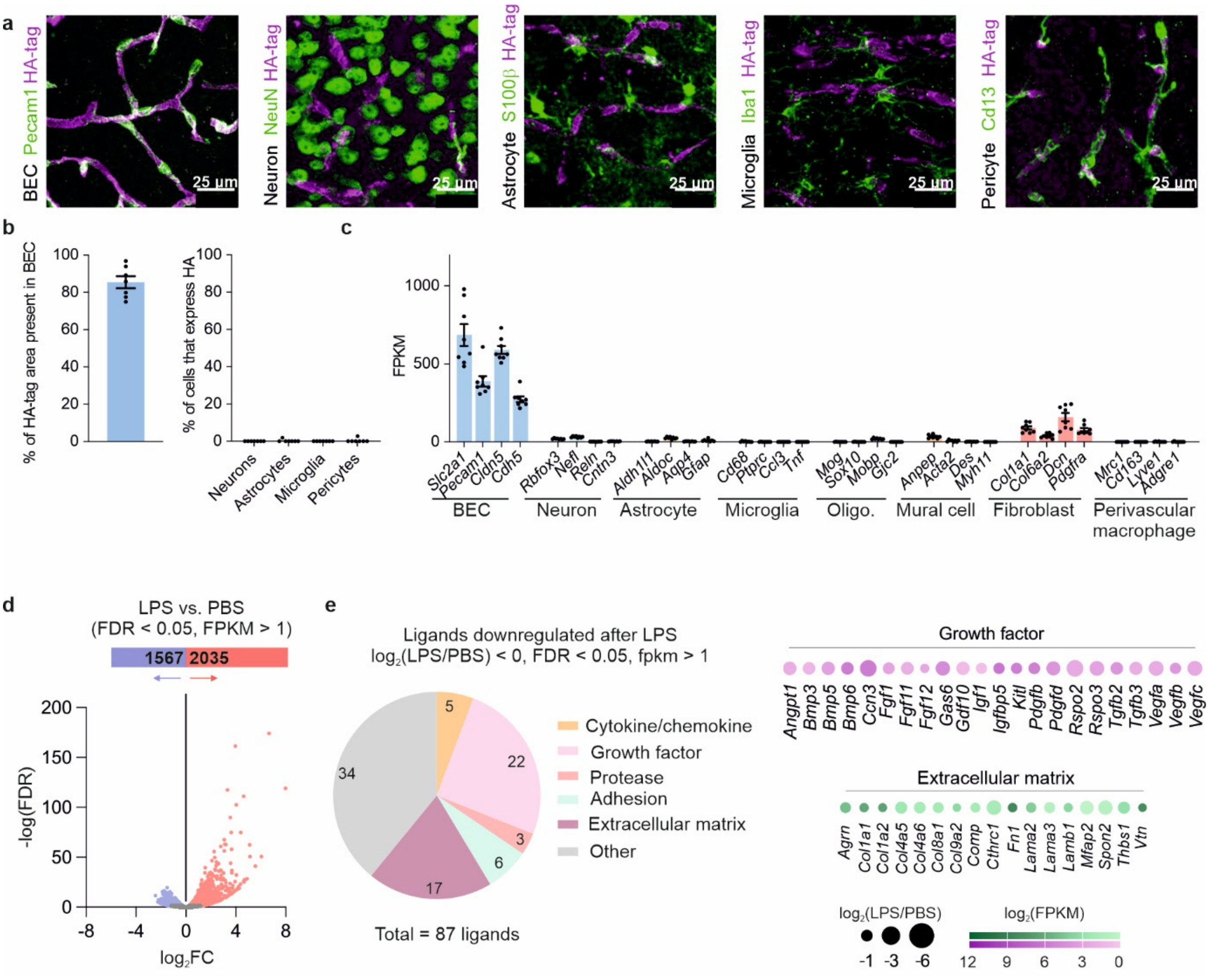

Supplementary figure 6

<u>Supplementary fig. 6.</u> Characterization of the *Cdh5-Ribotag* expression and RNA sequencing. **a:** Immunofluorescence of *Cdh5-Ribotag* mice after tamoxifen administration showing expression of Rpl22HA (HA-tag, magenta) in BECs (Pecam1), neurons (NeuN), astrocytes (S100β), microglia (Iba1), and pericytes (Cd13). Scale bars = 25 µm. **b:** Percent quantification of the HA-tag signal found in BECs (left) and other cell types (right). N = 3 PBS and 4 LPS mice, 2 images per mouse. Data are represented as mean ± SEM. Each dot represents the average data of all images per mouse. **c:** Graph of the expression levels in Fragments Per Kilobase of transcript per Million mapped reads (FPKM) of known cell-specific marker genes for BEC, neurons, astrocytes, microglia, oligodendrocytes, mural cells, fibroblasts and perivascular macrophages in RNA samples isolated from *Cdh5-Ribotag* mice. N = 4 PBS and 4 LPS mice. Data are represented as mean ± SEM. **d:** Volcano plot demonstrating differential expression in BECs with LPS vs. PBS. **e:** (Left) Classification of CellTalkDB ligands downregulated after LPS (total = 87 ligands). (Right) Dot plots for growth factors and ECM-related ligands downregulated after LPS. For dot plots, the color intensity of the dot corresponds to the abundance of the gene in LPS samples (Log_{10}FPKM), while the size of the dot corresponds to the log_2(LPS/PBS) of each gene. Proteins are referred to by their gene names. Source data are provided as a Source Data file and in Supplementary Data 4.

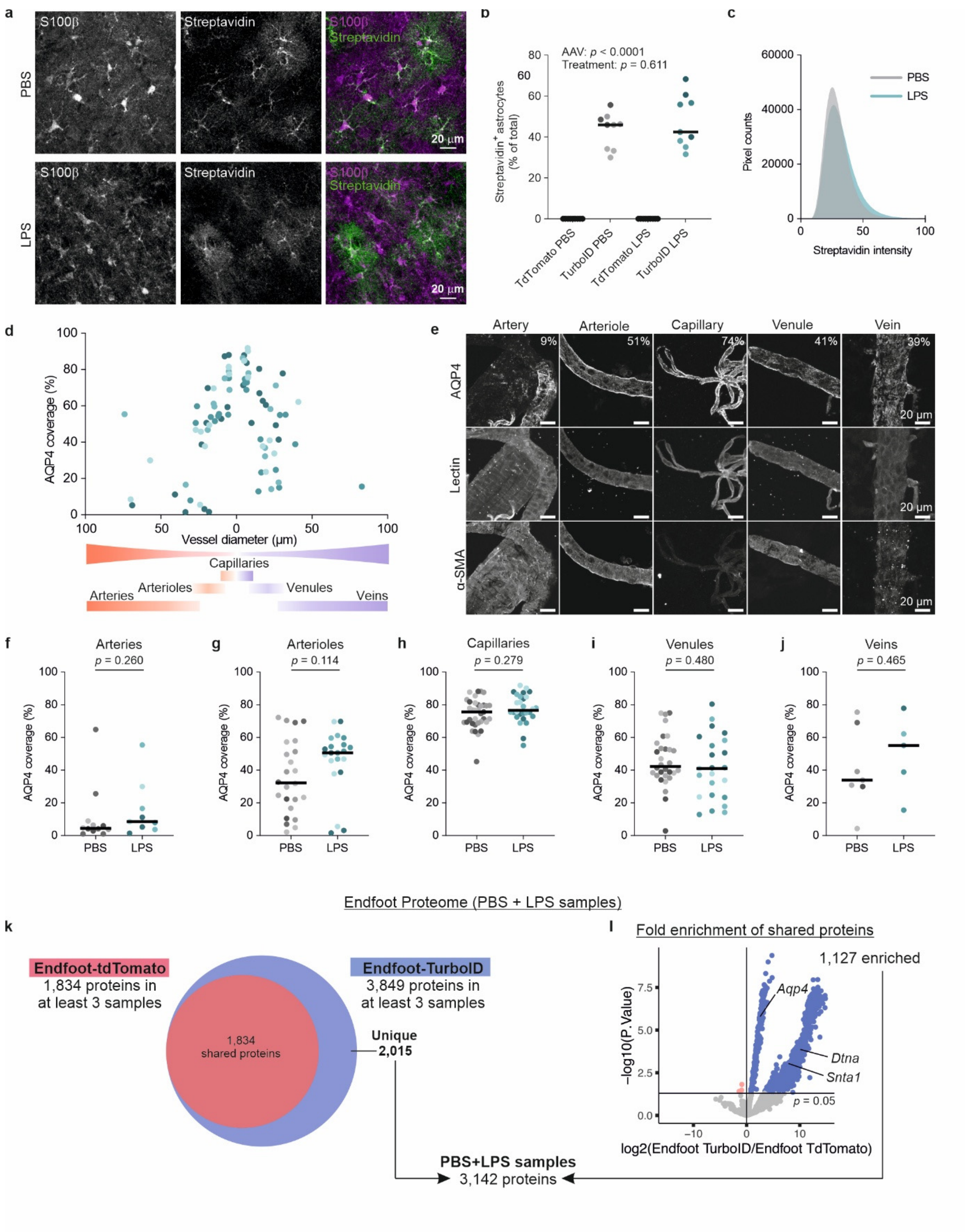

Supplementary figure 7

Supplementary fig. 7. Assessment of LPS potential effects on the astrocyte endfoot proteome method. a: Immunofluorescence for streptavidin and astrocytes (S100β) in mice receiving Astrocyte-TurboID AAV and PBS or LPS. Scale bars = 20 μm. **b:** Quantification of streptavidin-positive astrocytes after receiving Astrocyte-TurboID or Astrocyte-tdTomato. LMM was used to account for repeated measures per mouse: Intensity~AAV + Treatment + (1|Mouse), N = 2 mice for TdT PBS, N = 3 mice for all other conditions, 3 images/mouse; $F(1,8) = 101.85$ (AAV) and 0.280 (Treatment); no interaction between AAV and Treatment ($p = 0.667$). Horizontal lines represent the median; data points are shaded by mouse (PBS, grey; LPS, teal). **c:** Streptavidin intensity histograms from mice receiving PBS or LPS. Curves show average data of all images/treatment. N=3 mice, 3 images/mouse. **d:** Percent AQP4 coverage (lectin+ area colocalizing with AQP4) by vessel diameter in samples from mice receiving LPS. Dots left of 0 indicate arteries/arterioles; right of 0, venules/veins. Data points are shaded by mouse. N = 86 vessels from 4 mice, 19-24 vessels/mouse. Diagrams below represent the continuum of vessel diameter/type. **e:** Representative images of vessels in (d) stained with AQP4, lectin, and alpha-smooth muscle actin (α-SMA; striated smooth muscle lining arterioles/arteries). Percent AQP4 coverage indicated in first row. Scale bars = 20 μm. **f-j:** Percent AQP4 coverage in arteries (f), arterioles (g), capillaries (h), venules (i), and veins (j); (PBS, grey; LPS, teal). Horizontal lines represent the median. For each vessel type, LMM was used to account for repeated measures per mouse: Measurement~Treatment + (1|Animal), N = 5 mice for PBS, N = 4 mice for LPS, 1-7 vessels/condition/vessel-category/mouse; Arteries, $F(1,19) = 1.35$; Arterioles, $F(1,43) = 2.60$; Capillaries, $F(1,7) = 1.38$; Venules, $F(1,7.28) = 0.554$; Veins, $F(1,3.64) = 0.663$. Data points are shaded by mouse. **k:** Venn diagram comparing proteins in Endfoot-TurboID and Endfoot-tdTomato samples when data from LPS and PBS samples were combined. **l:** Volcano plot of shared proteins between Endfoot-TurboID and Endfoot-tdTomato. Known endfoot proteins are labeled. 1,127 enriched + 2,015 proteins unique in Endfoot-TurboID; total 3,142 proteins. Differential expression was performed using Limma. Proteins referred to by gene names. Exact sample sizes per animal and source data are provided in a Source Data file and Supplementary Data 5.

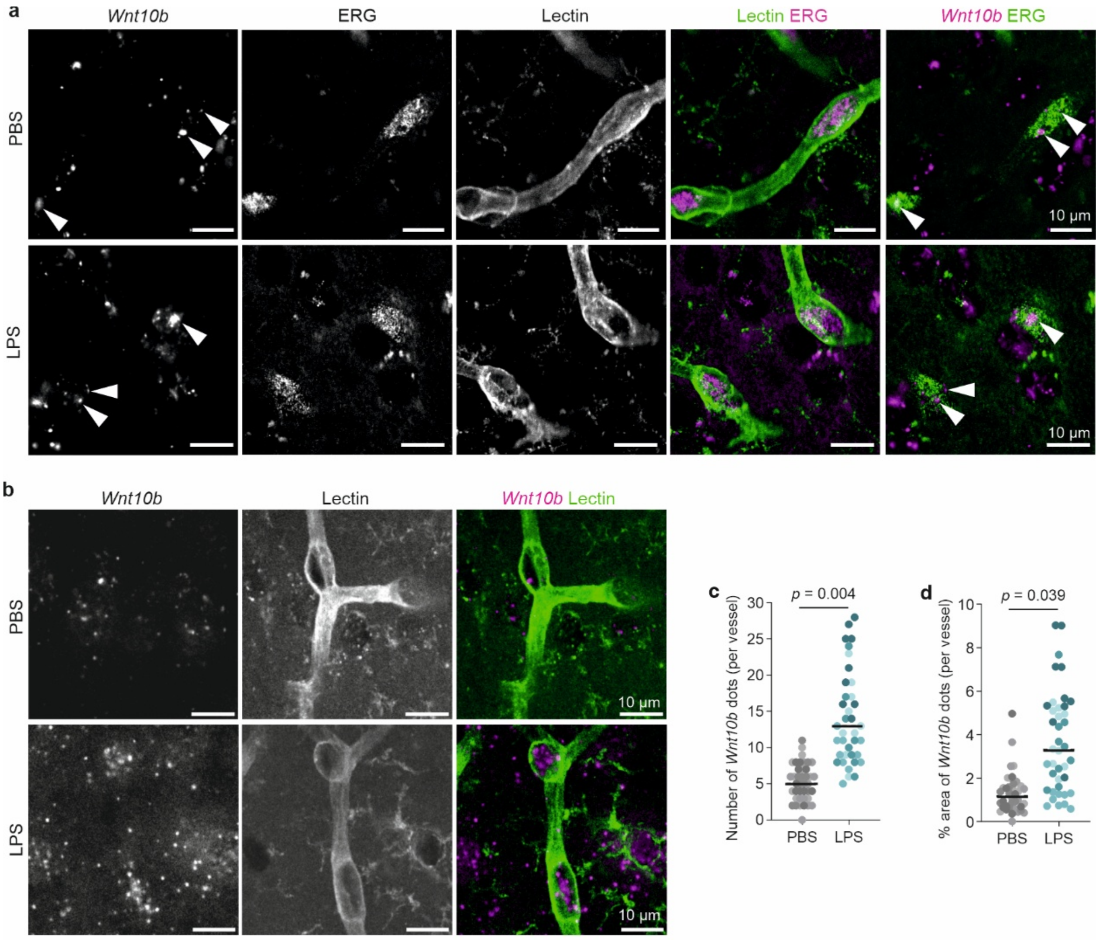

Supplementary figure 8

<u>Supplementary fig. 8.</u> Validation of increased *Wnt10b* expression in brain endothelial cells after LPS by RNAscope. a: Representative combined RNAscope and fluorescence-stained images of *Wnt10b* (RNAscope), BEC nuclei labeled with ERG (immunofluorescence staining), and blood vessels labeled with lectin (fluorescence staining) in cortex of mice receiving either PBS (top row) or LPS (bottom row). Arrowheads indicate *Wnt10b* dots associated with ERG. This experiment was performed in N = 4 mice per condition, with equivalent results**. b:** Representative combined RNAscope and fluorescence-stained images of *Wnt10b* (RNAscope - magenta) and blood vessels labeled with lectin (fluorescence staining - green) in cortex of mice receiving either PBS (top row) or LPS (bottom row). **c:** Quantification of the number of *Wnt10b* dots counted per vessel ROI stained with lectin after treatment of PBS or LPS. **d:** Graph indicating the percentage area labeled for *Wnt10b* within the vessel ROI. For data in graphs c-d, LMM was used to account for repeated measures from each mouse: Measurement ~ Treatment + (1 | Animal), N = 4 mice per condition, 10 vessels per mouse; for (c), $F(1,6) = 21.05$; for (d), $F(1,6) = 6.84$. In each graph, horizontal black lines represent the median, and data points from different mice are represented with different shades of grey (PBS) and teal (LPS). Scale bars = 10 µm. Source data are provided as a Source Data file.

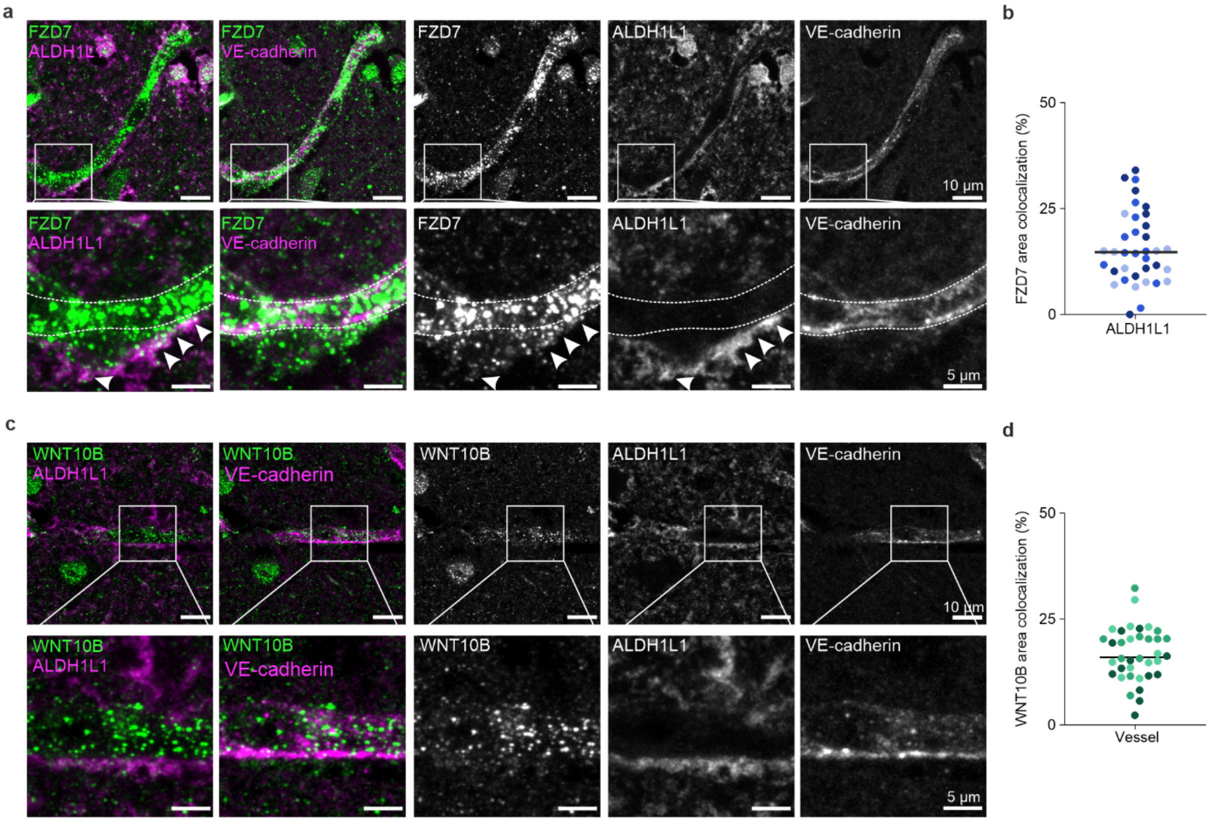

Supplementary figure 9

Supplementary fig. 9. Validation of FZD7 and WNT10B localization around blood vessels in human brain tissue. a: Representative immunofluorescence images from post-mortem human brain frontal cortex (BA46) with FZD7 (green), ALDH1L1 (magenta) and VE-cadherin (magenta). White insets indicate the region of each image displayed at higher magnification below. Arrowheads indicate areas of overlap between FZD7 and endfeet (ALDH1L1 positive signal surrounding the vessel) and the dotted line illustrates the boundary between vessels and endfeet. **b:** Quantification of the percentage area of ALDH1L1 positive signal surrounding the vessel that contains FZD7 signal. **c:** Representative immunofluorescence images from post-mortem human brain frontal cortex (BA46) immunolabeled with WNT10B (green), ALDH1L1 (magenta) and VE-cadherin (magenta). White insets indicate the region of each image displayed at higher magnification below. **d:** Quantification of the percentage area of vessel that contains WNT10B signal. The vessel area was defined by expanding the VE-cadherin ROI to include the endfoot region. N = 36 vessels from 3 individuals, 11-13 vessels per individual, with different color shades indicating vessels from each individual. Horizontal line indicates the median. Scale bars = 10 μm, or 5 μm in the insets. Source data are provided as a Source Data file.

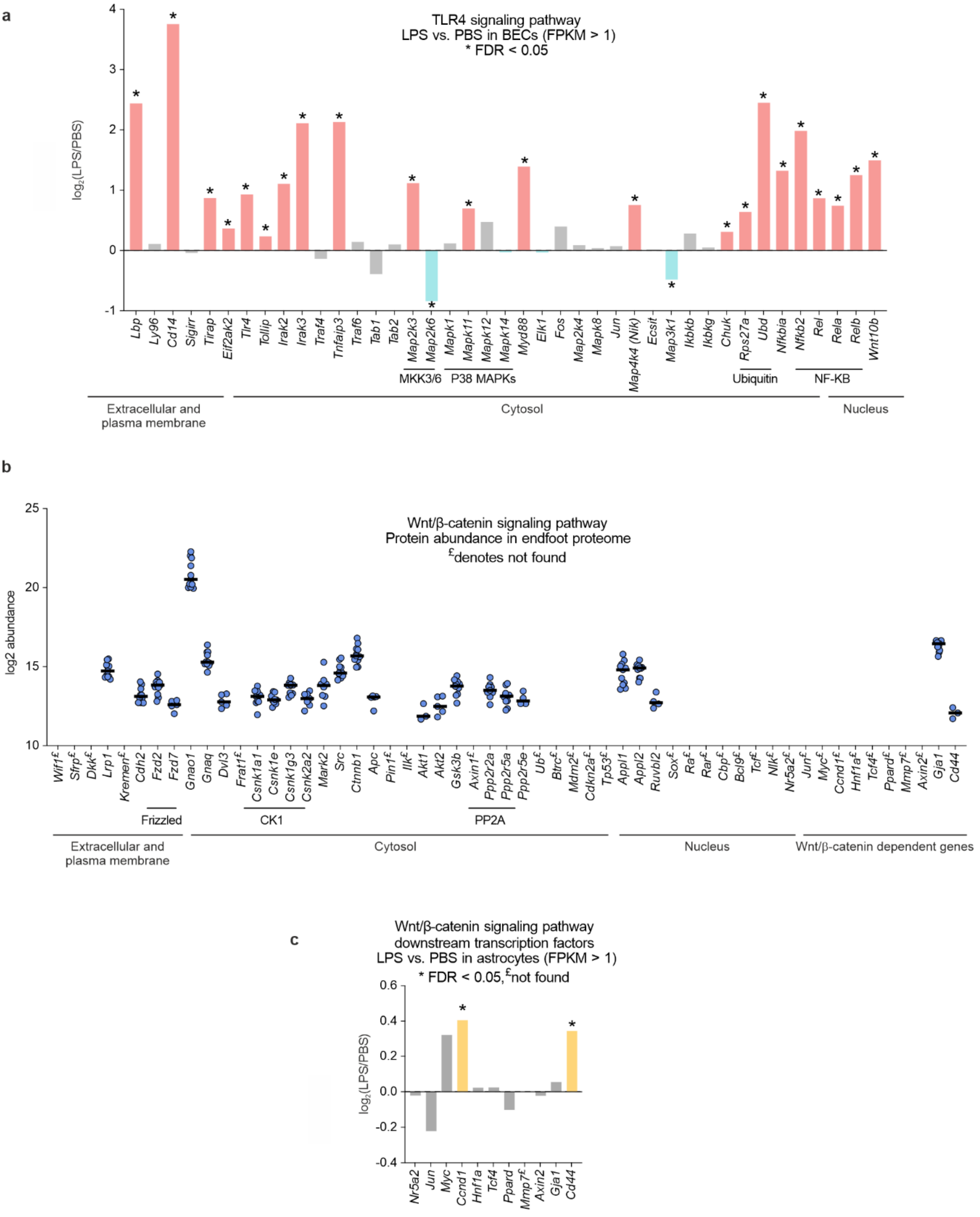

Supplementary figure 10

Supplementary fig. 10. Expression of WNT10B-FZD7 pathway components shown in Figure 6. a: Bars represent the log_2(LPS/PBS) of each gene from RNA sequencing of BECs after LPS (LPS vs. PBS, FPKM > 1, Supplementary Data 4). Red bars indicate genes that were significantly upregulated after LPS (FDR < 0.05). Light blue bars indicate genes that were significantly downregulated after LPS (FDR < 0.05). Grey bars indicate genes that were not significantly altered after LPS but were present in the dataset (FPKM > 1, FDR > 0.05). The cellular location for the protein corresponding to each gene is indicated below the graph. Where multiple genes correspond to a single protein in the schematic in Fig. 6i, the name in the schematic is indicated. Asterisks (*) indicate differentially expressed genes (LPS vs. PBS, FDR < 0.05). **b:** Graph of data used to generate the schematic in Fig. 6j. Each dot represents the log_2 abundance of each protein from an individual mouse (total N = 11 mice, Supplementary Data 5). Horizontal lines indicate median abundance values for each protein. The cellular location for each protein is indicated below the graph. Where multiple proteins correspond to a single molecule in the schematic in Fig. 6j, the name in the schematic is indicated. Asterisks (*) indicate proteins only found in TurboID samples or significant when compared to tdTomato controls (Supplementary Data 5). £ indicate proteins that were not found in the endfoot proteome dataset. The number of samples in which the protein has been detected varies depending on the proteins. **c:** Bar plot of data used to generate the genes activated by Wnt/β-catenin signaling in Fig. 6j (nucleus). Bars represent the log_2(LPS/PBS) of each gene from RNA sequencing of astrocytes[61] after LPS (LPS vs. PBS, FPKM > 1). Yellow bars indicate genes that were significantly upregulated in astrocytes after LPS (FDR < 0.05). Grey bars indicate genes that were not significantly altered after LPS but were present in the dataset (FPKM > 1, FDR > 0.05). Asterisks (*) indicate differentially expressed genes (LPS vs. PBS, FDR < 0.05). £ indicate genes that were not found in the dataset. Source data are provided as a Source Data file.

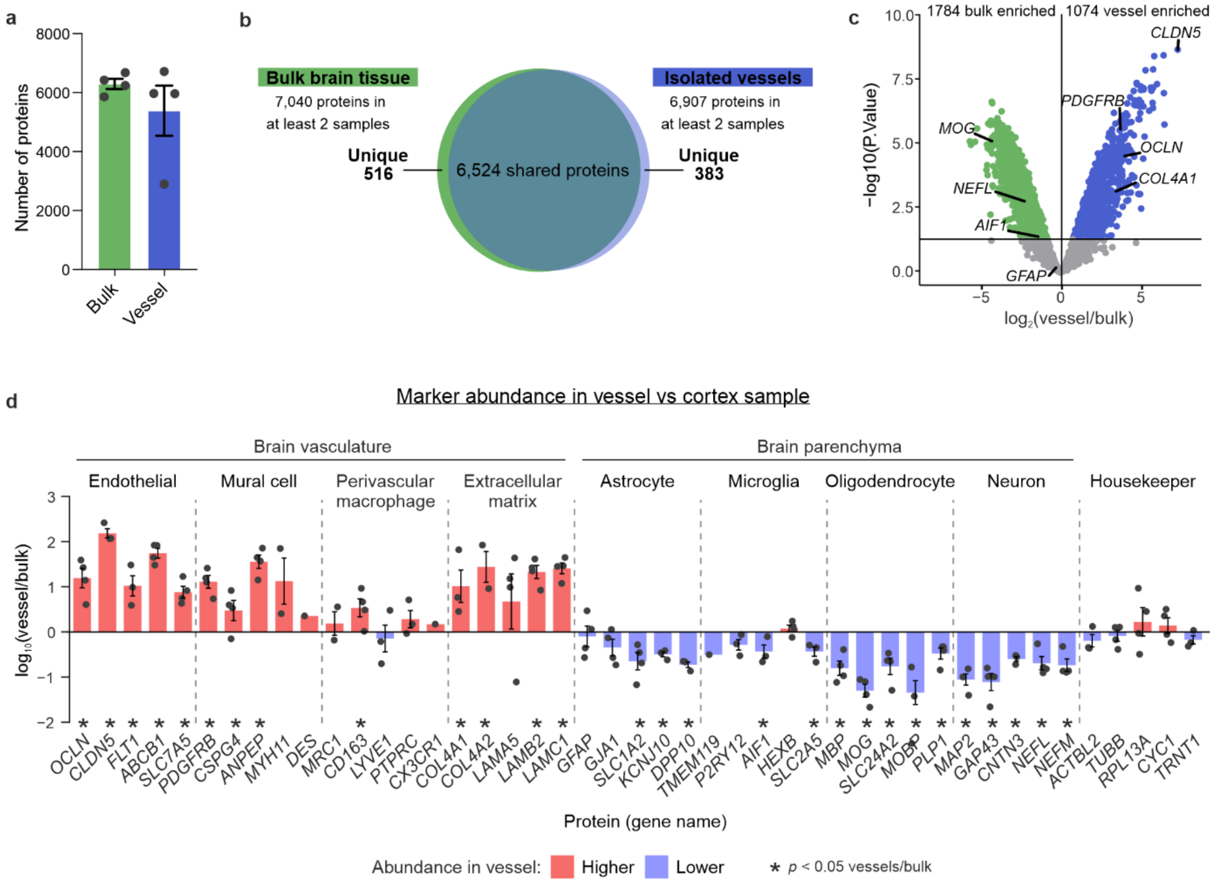

Supplementary figure 11

<u>Supplementary fig. 11.</u> Characterization of human vessel proteomics data. a: Bar graph of the number of proteins found in each sample type. Each dot represents one individual (N = 4; both sample types were derived from the same tissue sample). Data are represented as mean ± SEM. **b:** Venn diagram comparing proteins found in human bulk brain tissue and isolated vasculature. **c:** Volcano plot demonstrating the differential expression of shared proteins between isolated brain vasculature and bulk brain tissue in human cortex samples. Several cell-specific proteins are labeled. 1,074 proteins were enriched in isolated vasculature; 1,784 proteins were enriched in bulk brain tissue. **d:** Abundance of specific protein markers of the vasculature (endothelium, mural cell, perivascular macrophage, and basement membrane) and the brain parenchyma (astrocyte, microglia, oligodendrocyte, neuron) expressed as log10 fold-change (isolated vessel proteome/bulk cortex proteome). N = 4 cortical brain samples (see Table 2 in the Methods section for further information about the samples). Data are represented as mean ± SEM. Proteins are referred to by their gene names. * indicate significant difference in vessel vs bulk comparison with $p < 0.05$. Source data are provided as a Source Data file and in Supplementary Data 6.

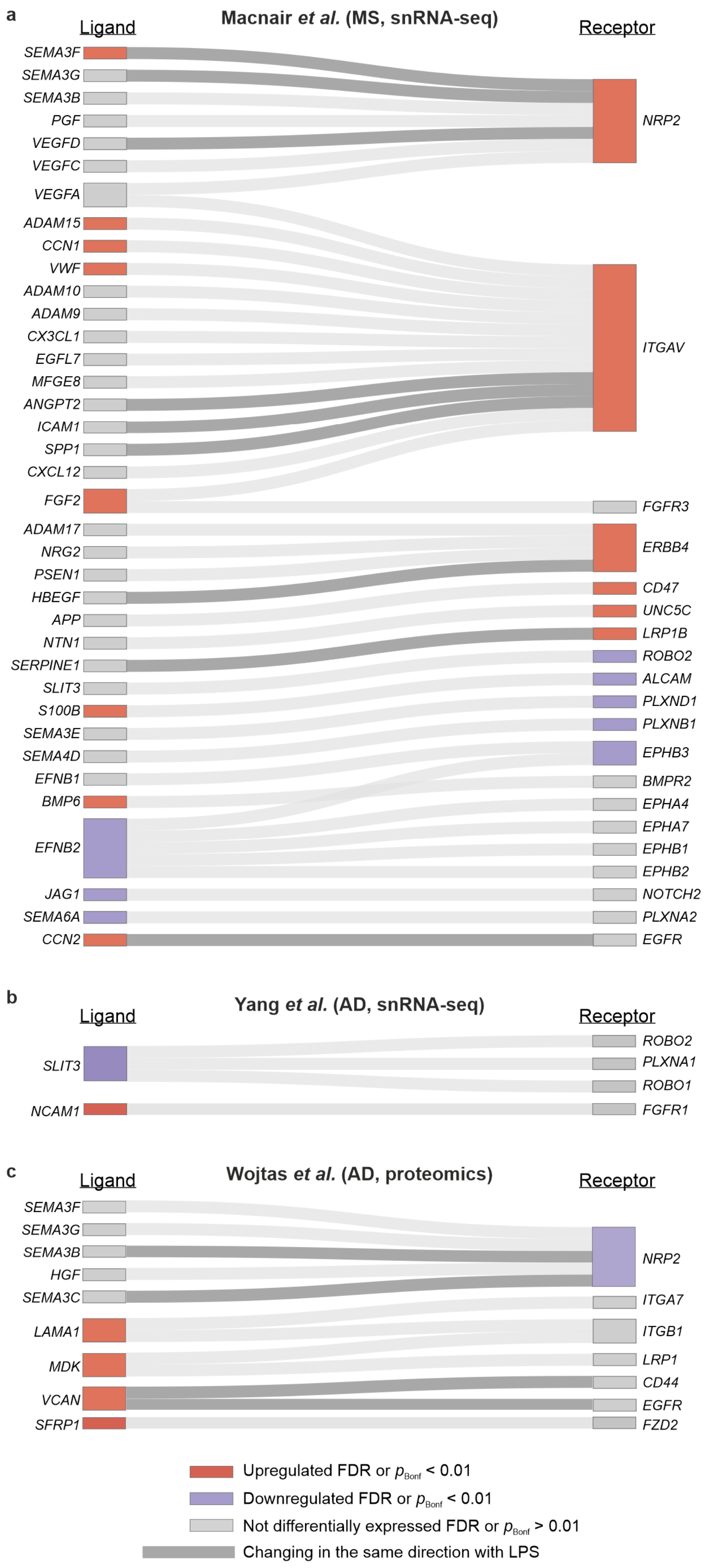

Supplementary figure 12

__Supplementary fig. 12.__ **Differential expression of mouse and human overlapping brain endothelial cell-endfoot ligand-receptor pairs in human neurodegenerative disease** (Fig. 6f and Supplementary Data 7). Upregulated proteins are indicated in red, downregulated in violet and not differentially expressed in grey for data from Macnair *et al.* (**a**), Yang *et al.* (**b**) and Wojtas *et al.* (**c**). Ligand-receptor pairs that changed in the same direction in mice injected with LPS are highlighted with connecting lines in dark grey. Proteins are referred to by their gene names.
