# Supplementary material for: Molecular profiling of brain endothelial cell to astrocyte endfoot communication in mouse and human
Source: Nat Commun. 2025 Nov 6;16:9750. doi: 10.1038/s41467-025-65487-4 (PMC12592424; doi:10.1038/s41467-025-65487-4)
Supplement: Supplementary file 2 — Description of Additional Supplementary Files [file 41467_2025_65487_MOESM2_ESM.docx]

**Description of Additional Supplementary Files**

**Supplementary Data 1:** Astrocyte proteome. Excel file contains all identified proteins, protein abundances, and differential expression (TurboID vs. tdTomato). A readme tab contains relevant analysis and sample information.

**Supplementary Data 2:** Endfoot proteome and IPA pathways. Excel file contains all identified proteins, protein abundances, and differential expression (TurboID vs. tdTomato) for the endfoot proteome and comparisons between endfoot and astrocyte proteomes (EF vs. Astro). IPA pathways represented in the endfoot proteome are also included. A readme tab contains relevant analysis and sample information.

**Supplementary Data 3:** Endfoot proteome comparisons with other endfoot datasets. Labeled tabs contain proteins found in the listed comparisons. A readme tab contains information on datasets used for comparison, including DOI, brain region, and any edits to the data prior to comparison.

**Supplementary Data 4:** Brain endothelial cell RNA seq data and pathways. Excel file contains differential expression (LPS_vs_PBS, IP), corresponding IPA pathways, and unfiltered RNA seq data. A readme tab contains relevant analysis and sample information.

**Supplementary Data 5:** Endfoot proteome with LPS and PBS samples. Excel file contains all identified proteins, protein abundances, and differential expression for the endfoot proteome when samples are combined (LPS+PBS, TurboID vs. tdTomato) and compared between treatments (LPS vs PBS, TurboID). IPA pathways represented in the LPS vs PBS comparison are also included. A readme tab contains relevant analysis and sample information.

**Supplementary Data 6:** Human proteome for isolated cortical vessels and bulk tissue. Excel file contains sample information, all identified proteins, protein abundances, and differential expression (vessel vs bulk). A readme tab contains relevant analysis information.

**Supplementary Data 7:** Ligand-receptors pairs in mouse and human datasets. Excel file contains all identified ligand-receptor pairs in mouse and their presence in human proteomics data from this study and other published datasets. A readme tab contains relevant analysis and sample information.
