# Supplementary material for: Molecular profiling of brain endothelial cell to astrocyte endfoot communication in mouse and human
Source: Nat Commun. 2025 Nov 6;16:9750. doi: 10.1038/s41467-025-65487-4 (PMC12592424; doi:10.1038/s41467-025-65487-4)
Supplement: Supplementary file 10 — Reporting Summary [file 41467_2025_65487_MOESM10_ESM.pdf]

nature portfolio

Corresponding author(s): Blanca Díaz Castro

Last updated by author(s): May 2, 2025

Reporting Summary

Nature Portfolio wishes to improve the reproducibility of the work that we publish. This form provides structure for consistency and transparency in reporting. For further information on Nature Portfolio policies, see our Editorial Policies and the Editorial Policy Checklist.

Statistics

For all statistical analyses, confirm that the following items are present in the figure legend, table legend, main text, or Methods section.

n/a	Confirmed	
☐	☒	The exact sample size (*n*) for each experimental group/condition, given as a discrete number and unit of measurement
☐	☒	A statement on whether measurements were taken from distinct samples or whether the same sample was measured repeatedly
☐	☒	The statistical test(s) used AND whether they are one- or two-sided<br>*Only common tests should be described solely by name; describe more complex techniques in the Methods section.*
☐	☒	A description of all covariates tested
☐	☒	A description of any assumptions or corrections, such as tests of normality and adjustment for multiple comparisons
☐	☒	A full description of the statistical parameters including central tendency (e.g. means) or other basic estimates (e.g. regression coefficient) AND variation (e.g. standard deviation) or associated estimates of uncertainty (e.g. confidence intervals)
☐	☒	For null hypothesis testing, the test statistic (e.g. *F*, *t*, *r*) with confidence intervals, effect sizes, degrees of freedom and *P* value noted<br>*Give P values as exact values whenever suitable.*
☒	☐	For Bayesian analysis, information on the choice of priors and Markov chain Monte Carlo settings
☐	☒	For hierarchical and complex designs, identification of the appropriate level for tests and full reporting of outcomes
☒	☐	Estimates of effect sizes (e.g. Cohen's *d*, Pearson's *r*), indicating how they were calculated

Our web collection on statistics for biologists contains articles on many of the points above.

Software and code

Policy information about availability of computer code

Data collection	Proteomics: for mouse, peptides were analyzed on a Bruker timsTOF SCP mass spectrometer, and data were acquired in standard DIA PASEF template mode. For human proteomics, peptides were analyzed on an Orbitrap Fusion Lumos Tribrid mass spectrometer in data independent acquisition (DIA) mode.<br><br>RNA-seq: The concentration and quality of the RNA was assessed with an Agilent 2100 Bioanalyzer and only RNA samples with a concentration higher than 4 µg/µL and a RNA integrity number (RIN) greater than 7 were further sequenced. Sequencing libraries were prepared using TruSeq RNA stranded mRNA kit (Illumina Cat# 20020594) and libraries were sequenced on a NextSeq 500 system (Illumina).
Data analysis	Raw mass spectra files were analyzed using DIA-NN v.1.8.1 in library free mode (DOI: https://doi.org/10.1038/s41592-019-0638-x). For mouse samples, peptides were searched against a Uniprot mouse database (downloaded March 2022) supplemented with TurboID and tdTomato sequences. For human samples, peptides were searched against a Uniprot reference human proteome downloaded March 2023. Both mouse and human peptides were also searched against a custom database of common contaminants based on the cRAP database (https://www.thegpm.org/crap/). The default parameters for double pass neural network classifier were used with the following exceptions: the mass accuracy and MS1 accuracy was set to 10.0, and "Heuristic protein inference" was unselected. Precursor false discovery rate was set to 0.1.<br><br>All proteomics data presented here originates from the "report.pg_matrix.tsv" output file from DIA-NN. All analysis was conducted using R (R Core Team, 2023), RStudio (RStudio Team, 2023), and the tidyverse package (DOI: 10.21105/joss.01686).<br>Ambiguous, species-mismatched, contaminant proteins, and proteins not mapped to genes were removed from analysis. For comparisons between mice expressing TurboID and tdTomato AAV, proteins found in at least 33% of the samples from either astrocyte or endfoot protein isolations regardless of AAV were kept for further analysis due to the inherent difference in isolated proteins between TurboID and tdTomato samples. For comparisons between LPS and PBS samples, which only received TurboID AAV and are therefore expected to yield a similar

number of isolated proteins between conditions, proteins were kept for further analysis if they were found in at least 50% of both PBS and LPS samples. Human proteins were kept if they appeared in at least 2 samples from bulk or at least 2 samples from vessel protein isolations. Quantile normalisation was performed using the preprocessCore package v1.66 (https://www.bioconductor.org/packages/release/bioc/html/preprocessCore.html). Mouse astrocyte and endfoot datasets were normalised separately, human bulk and vessel datasets were normalised together. For mouse samples, differential expression analysis was conducted with the package limma v3.56.2 (https://www.bioconductor.org/packages/release/bioc/html/limma.html). Differential expression was considered significant when p-value < 0.05. For human samples, differential expression analysis was conducted with the package DEP v1.26.0 (https://bioconductor.org/packages/release/bioc/html/DEP.html). Differential expression was considered significant when adjusted p-value < 0.05 with a log2 fold-change cutoff of ± 1. Gene ontology analysis was performed using Enrichr (https://maayanlab.cloud/Enrichr/), and further analysis was performed using Ingenuity Pathway Analysis (Qiagen).

RNA-seq: Reads were mapped to the primary assembly of the mouse reference genome contained in Ensembl release, using the STAR RNA-seq aligner, version 2.7.9a. Tables of per-gene read counts were then generated from the mapped reads with featureCounts, version 2.0.2. Differential gene expression was performed in R using DESeq2, version 1.30.1.

Line analyses of immunofluorescense signal in crosssections of vessels was quantified using Clampfit (Molecular Devices). Graphs were generated using both GraphPad Prism and R. Most graphs generated in R were made with the ggplot2 package (version 3.5.1). Sankey plots in Figures 4, 7, and Supplementary Fig. 12 were made using the networkD3 (version 0.4) and htmlwidgets (version 1.6.4) packages. UpSet plots in Figures 1, 8, and Supplementary Fig. 3 were made using the UpSetR package (version 1.4.0). Circle dendrogram in Supplementary Fig. 4 was generated the ggraph (version 2.2.1) and igraph (version 2.1.2) packages.

For manuscripts utilizing custom algorithms or software that are central to the research but not yet described in published literature, software must be made available to editors and reviewers. We strongly encourage code deposition in a community repository (e.g. GitHub). See the Nature Portfolio guidelines for submitting code & software for further information.

Data

Policy information about availability of data

All manuscripts must include a data availability statement. This statement should provide the following information, where applicable:

- Accession codes, unique identifiers, or web links for publicly available datasets
- A description of any restrictions on data availability
- For clinical datasets or third party data, please ensure that the statement adheres to our policy

The mass spectrometry proteomics data have been deposited to the ProteomeXchange Consortium via the PRIDE partner repository with the dataset identifier PXD056232 (https://www.ebi.ac.uk/pride/archive/projects/PXD056232) for the mouse dataset, and identifier PXD055837 (https://www.ebi.ac.uk/pride/archive/projects/PXD055837) for the human dataset.

RNA-seq data was uploaded onto ArrayExpress with accession ID E-MTAB-14490 (https://www.ebi.ac.uk/biostudies/arrayexpress/studies/E-MTAB-14490).

Proteomics and RNA-seq data can be explored at https://neuromics-explorer.ukdri.ac.uk/.

All data analyzed in this study are provided in Extended data tables 1-7 and the source data file.

Source data are provided with this paper.

Research involving human participants, their data, or biological material

Policy information about studies with human participants or human data. See also policy information about sex, gender (identity/presentation), and sexual orientation and race, ethnicity and racism.

Reporting on sex and gender	This study used human post-mortem brain tissue from 4 female and 5 male individuals. Sex information is based on clinical data. No sex-based analyses have been conducted dure to lack of enough statistical power.
Reporting on race, ethnicity, or other socially relevant groupings	Race and ethnicity information has not been recorded by the brain bank provisioning the human tissue for this study. Therefore, no information is reported nor used for analysis.
Population characteristics	All human tissue used in this study was obtained from individuals between 30-45 years without neurological disease. Further information regarding human samples is listed in Extended Data Table 5.
Recruitment	No direct recruitment was performed in this study.
Ethics oversight	The tissue was obtained with full ethical approval from the Edinburgh Brain and Tissue Bank which has ethical approval from the East of Scotland Research Ethics Service REC1 to function as a research tissue bank. Informed consent was either obtained from participants in life and/or from the nearest relative for deceased participants, in keeping with legal requirements. There is no compensation or financial benefit to participants or their families.

Note that full information on the approval of the study protocol must also be provided in the manuscript.

Field-specific reporting

Please select the one below that is the best fit for your research. If you are not sure, read the appropriate sections before making your selection.

☒ Life sciences ☐ Behavioural & social sciences ☐ Ecological, evolutionary & environmental sciences

For a reference copy of the document with all sections, see nature.com/documents/nr-reporting-summary-flat.pdf

Life sciences study design

All studies must disclose on these points even when the disclosure is negative.

Sample size	For proteomics, samples sizes were chosen based on previously published related research (DOI: 10.1038/s41586-023-05927-7; DOI: 10.1038/s41586-020-2926-0). The samples sizes chosen where sufficient to detect differences between conditions.<br><br>For RNA-seq, the sample size was based on previously published results using similar conditions (DOI: 10.1016/j.celrep.2021.109508).<br>No sex-based analyses have been conducted dure to lack of enough statistical power.<br><br>For immunofluorescence studies, all quantification except Fig. 1i, were anbalysed with liner mixed-effects models. Where a large effect size was expected a n = 3-4 mice and 3 images per mouse was used, e.g. Supp. Fig. 1. Where smaller effect sizes were expected, n = 4-6 mice and 5-10 images per mouse were used, e.g. Fig. 6. Similar n number have been used in similar studies (doi: 10.1038/s41586-020-2926-0; doi: 10.1038/s41586-023-05927-7; doi: 10.1016/j.neuron.2025.05.019; doi: 10.1038/s41467-025-63131-9)
Data exclusions	One case each was excluded from further analysis in both mouse and human proteomics due to technical failure (less than 50% of normally identified proteins were identified in the endfoot or vessel proteome, indicating poor quality sample preparation).
Replication	All data collection was done in multiple experiments. Mouse proteomics experiments were conducted in batches of 5 mice per isolation in a total of 4 separate isolations. All astrocyte and endfoot samples are biological replicates within the sample group but are technical replicates across groups (i.e., each astrocyte protein sample has a pair endfoot sample taken from the same mouse brain). Human proteins were isolated in a single batch. RNA-seq samples were isolated in batches of up to 8 mice, and each sample RNA-seq sample contains pooled RNA from two mice. All experiments were successfully replicated.
Randomization	For all mouse experiments, mice were purchased from the supplier and each cage was randomly assigned to an experimental group.<br><br>For mouse proteomics, samples were prepared in batches that contained one sample of each group in each batch to avoid batch effects being associated to specific groups. Samples were run in the mass spectrometer in random order.<br><br>For human proteomics, all samples represent the same experimental group and therefore could not be randomized.<br><br>For RNA-seq, mice were bred in house and animals randomly assigned to each experimental group. Samples were prepared in batches that contained the same number of samples of each group in each batch to avoid batch effects being associated to specific groups.
Blinding	For proteomics and RNA-seq, the operators ofmass spectrometers and sequencers were blinded. The investigators that collected and procesed the samples were not blinded to the conditions of study, to be able to avoid batch effects (see above).<br><br>For confocal image acquisition and analysis in FIJI/ImageJ, investigators were blinded to the experimental identify of the samples.

Reporting for specific materials, systems and methods

We require information from authors about some types of materials, experimental systems and methods used in many studies. Here, indicate whether each material, system or method listed is relevant to your study. If you are not sure if a list item applies to your research, read the appropriate section before selecting a response.

Materials & experimental systems

n/a	Involved in the study	
☐	☒	Antibodies
☐	☒	Eukaryotic cell lines
☒	☐	Palaeontology and archaeology
☐	☒	Animals and other organisms
☒	☐	Clinical data
☒	☐	Dual use research of concern
☒	☐	Plants

Methods

n/a	Involved in the study	
☒	☐	ChIP-seq
☒	☐	Flow cytometry
☒	☐	MRI-based neuroimaging

Antibodies

Antibodies used

Primary antibodies:
rabbit anti-S100β (1:1000, Abcam Cat# ab41548)
mouse anti-S100β (1:1000, Sigma Cat# S2532)
guinea pig anti-S100β (1:500, Synaptic Systems Cat# 287004)
mouse anti-NeuN (1:500, Sigma Cat# MAB377)
rabbit anti-NeuN (1:2000, Cell Signaling Cat# 12943S)
rabbit anti-RFP (1:1000, Rockland Cat# 600-401-379)
goat anti-IBA1 (1:1000, Abcam Cat# ab5076)
rabbit anti-IBA1 (1:1000, Wako Cat# 019-19741)
rabbit anti-aquaporin4 (1:1000, Millipore Cat# ab3594)
mouse anti-aquaporin4 (1:100, Abcam, Cat# ab9512)
mouse anti-β-dystroglycan (1:1000, DSHB Cat# 7D11)
rabbit anti-HA (1:500, Cell Signaling, Cat# C29F4)
rat anti-PECAM1 (1:50, BD Biosciences Cat# 550274)
goat anti-CD13 (1:100, R&D Cat# AF2335)
mouse anti-CC1 (1:300, Sigma Cat# OP80)
rabbit anti-Fzd7 (1:500, Abcam Cat# ab64636)
rabbit anti-Frizzled7 (1:250, Proteintech, 16974-1-AP)
rabbit anti-Wnt10b (1:200, Abcam Cat# ab70816)
rabbit anti-PYGB (1:500, Atlas antibodies Cat# HPA031067)
mouse anti-ALDH1L1 (1:200, NeuroMab Cat# 75-140)
goat anti-VE-cadherin (1:200, R&D Systems Cat# AF938)
biotinylated lectin (1:500, Vector Cat# B-1175-1)
rabbit anti-ERG (1:500, abcam, Cat# ab92513)
fluorescein-labeled lectin 1:200 (Vector Cat# 1171)
chicken anti-GFP (1:1000, Abcam, Cat# ab13970)
mouse anti-α-Smooth Muscle Actin (ACTA2) (1: 200, Sigma-Aldrich Cat#A5228)

Secondaries (all 1:1000 unless indicated):
goat anti-mouse IgG Alexa 488 (Invitrogen Cat# A11001)
goat anti-rabbit IgG Alexa 488 (Invitrogen Cat# A11008)
donkey anti-mouse IgG Alexa 488 (Invitrogen Cat# A21202)
donkey anti-rabbit IgG Alexa 488 (Invitrogen Cat# A21206)
goat anti-rabbit IgG Alexa 546 (Invitrogen Cat# A11010)
donkey anti-rabbit IgG Alexa 546 (Invitrogen, Cat# A10040)
donkey anti-rat IgG Cy3 (Jackson Immuno Research Cat# 712-165-150)
goat anti-mouse 546 (Invitrogen, Cat# A11003)
goat anti-mouse IgG Alexa 647 (Invitrogen Cat# A21235)
goat anti-rabbit IgG Alexa 647 (Invitrogen Cat# A21244)
goat anti-rat IgG Alexa 647 (Invitrogen Cat# A21247)
donkey anti-goat IgG Alexa 647 (Invitrogen Cat# A21447)
conjugated streptavidin Alexa 405 (Invitrogen, Cat# S32351)
conjugated streptavidin Alexa 488 (1:200, Invitrogen Cat# S32354)
conjugated streptavidin Alexa 405 (Invitrogen, Cat# S32351)
goat anti-rabbit 488 (Invitrogen, Cat# A11008)
goat anti-chicken Alexa 488 (Invitrogen, Cat# A11039)

Validation

rabbit anti-S100β (Abcam Cat# ab41548) has been validated in mouse brain in publications such as Brown et al, 2024 (doi: 10.3389/fncel.2023.1094503)
mouse anti-S100β (Sigma Cat# S2532) is validated by Sigma for immunofluorescence in mouse tissue.
guinea pig anti-S100β (Synaptic Systems Cat# 287004) is validated by Synaptic Systems for immunofluorescence in mouse tissue.
mouse anti-NeuN (Sigma Cat# MAB377) is validated by Merck for immunofluorescence in mouse tissue.
rabbit anti-NeuN (Cell Signaling Cat# 12943S) is validated by Cell Signaling for immunofluorescence in mouse tissue.
rabbit anti-RFP (Rockland Cat# 600-401-379) is validated by Rockland for immunofluorescence in mouse tissue.
goat anti-IBA1 (Abcam Cat# ab5076) has been used with mouse brain in multiple publications, such as Miao et al, 2023 (doi: 10.14336/AD.2023.0516-1).
rabbit anti-IBA1 (Wako Cat# 019-19741) has been used with mouse brain in multiple publications, such as Sakakibara et al 2019, doi: 10.1186/s12868-019-0496-6).
rabbit anti-aquaporin4 (Millipore Cat# ab3594) has been used with mouse brain in multiple publications, such as Hablitz et al 2020, doi: 10.1038/s41467-020-18115-2.
mouse anti-aquaporin4 (1:100, Abcam, Cat# ab9512) has been used with mouse brain in multiple publications, such as doi.org/10.1172/JCI169131.
mouse anti-b-dystroglycan (DSHB Cat# 7D11) has been used in multiple publications such as DOI: 10.1074/jbc.M112.400044.
rabbit anti-HA (Cell Signaling, Cat# C29F4) is validated for immunoprecipitation by the manufacturer.
rat anti-PECAM1 (BD Biosciences Cat# 550274) has been used with mouse brain in multiple publications, such as Kryzwinska et al 2017, doi: 10.1038/s41467-017-01599-w.

goat anti-CD13 (R&D Cat# AF2335) has been used with mouse brain in multiple publications, such as Zhao et al 2022, doi: 10.1073/pnas.2113310119.
mouse anti-CC1 (Sigma Cat# OP80) is validated as suitable for immunofluorescence by the manufacturer.
rabbit anti-Fzd7 (Abcam Cat# ab64636) is validated as suitable for immunofluorescence in mouse and human tissue by the manufacturer.
rabbit anti-Frizzled7 (1:250, Proteintech, 16974-1-AP) is validated as suitable for immunofluorescence in mouse and human tissue by the manufacturer.
rabbit anti-Wnt10b (Abcam Cat# ab70816) has been used in mouse in publications such as DOI: 10.1155/2020/9451596 and has been validated as suitable for immunofluorescence in human tissue by the manufacturer.
rabbit anti-PYGB (Atlas antibodies Cat# HPA031067) has been used in mouse in publications such as DOI: 10.3389/fnmol.2021.819440
mouse anti-ALDH1L1 (NeuroMab Cat# 75-140) is validated as suitable for human immunofluorescence by the manufacturer.
goat anti-VE-cadherin (R&D Systems Cat# AF938) is validated as suitable for human immunofluorescence by the manufacturer.
biotinylated lectin (Vector Cat# B-1175-1) is validated as suitable for vascular endothelium labelling in mouse by the manufacturer.
fluorescein-labeled lectin 1:200 (Vector Cat# 1171) is validated as suitable for vascular endothelium labelling in mouse by the manufacturer.
rabbit anti-ERG (1:500, abcam, Cat# ab92513) is validated as suitable for labelling of nuclei of brain endothelial cells in mouse by the manufacturer and hundreds of publications (some of themlisted at the manufacture's website.
chicken anti-GFP (1:1000, Abcam, Cat# ab13970) is validated as suitable for green fluorescent protein (GFP) labelling by the manufacturer.
mouse anti-α-Smooth Muscle Actin (ACTA2) (1: 200, Sigma-Aldrich Cat#A5228) is validated as suitable for alpha smooth muscle actin protein labelling by the manufacturer and published in manuscritps like doi.org/10.1016/j.isci.2023.108010

Eukaryotic cell lines

Policy information about cell lines and Sex and Gender in Research

Cell line source(s)	HEK 293T cells ((ATCC)293T/17 CRL-11268)
Authentication	We purchased the HEK-293T cells from the ATCC: CRL 3216
Mycoplasma contamination	Mycoplasma negative cell. AAV mycoplasma detection assay: Lower than 0.5EU/mL: We are release AAV batches lower than 0.5EU/mL.
Commonly misidentified lines (See ICLAC register)	*Name any commonly misidentified cell lines used in the study and provide a rationale for their use.*

Animals and other research organisms

Policy information about studies involving animals; ARRIVE guidelines recommended for reporting animal research, and Sex and Gender in Research

Laboratory animals	Most experiments were performed using C57Bl/6J mice (Charles River Laboratories, UK) with male and female mice in equal proportion. For brain endothelial cell RNAseq experiments, Cdh5-Cre/ERT2 (Tg(Cdh5-cre/ERT2)1Rha) mice were bred with B6N.129-Rpl22tm1.1Psam/J (RRID: IMSR_JAX: 011029). For RNA-seq experiments, mice were 12-16 weeks of age. For proteomics experiments, mice were 22 weeks of age.
Wild animals	No wild animals were used in this study.
Reporting on sex	Male and female mice were used in equal proportion for all experiments.
Field-collected samples	No field-collected samples were used in this study.
Ethics oversight	All procedures were regulated and approved by the UK Home Office under Project Licence PP8310627. All experiments were conducted under the UK Home Office Animals (Scientific Procedures) Act 1986, in agreement with local ethical and veterinary approval (Biomedical Research Resources, University of Edinburgh). All investigators working with mice held a Personal Licence from the UK Home Office.<br>Human tissue samples were obtained with full ethical approval from the Edinburgh Brain and Tissue Bank which has ethical approval from the East of Scotland Research Ethics Service REC1 to function as a research tissue bank.

Note that full information on the approval of the study protocol must also be provided in the manuscript.

Plants

Seed stocks	n/a
Novel plant genotypes	n/a
Authentication	n/a
